# Social exclusion modulates priorities of attention allocation in cognitive control

**DOI:** 10.1038/srep31282

**Published:** 2016-08-11

**Authors:** Mengsi Xu, Zhiai Li, Liuting Diao, Lijie Zhang, Jiajin Yuan, Cody Ding, Dong Yang

**Affiliations:** 1 School of Psychology, Southwest University, Chongqing, China; 2 Key Laboratory of Cognition and Personality (Southwest University), Ministry of Education, Chongqing, China; 3 The School of Psychology and Cognitive Science, East China Normal University, Shanghai, China; 4 University of Missouri-St. Louis, St. Louis, USA

## Abstract

Many studies have investigated how exclusion affects cognitive control and have reported inconsistent results. However, these studies usually treated cognitive control as a unitary concept, whereas it actually involved two main sub-processes: conflict detection and response implementation. Furthermore, existing studies have focused primarily on exclusion’s effects on conscious cognitive control, while recent studies have shown the existence of unconscious cognitive control. Therefore, the present study investigated whether and how exclusion affects the sub-processes underlying conscious and unconscious cognitive control differently. The Cyberball game was used to manipulate social exclusion and participants subsequently performed a masked Go/No-Go task during which event-related potentials were measured. For conscious cognitive control, excluded participants showed a larger N2 but smaller P3 effects than included participants, suggesting that excluded people invest more attention in conscious conflict detection, but less in conscious inhibition of impulsive responses. However, for unconscious cognitive control, excluded participants showed a smaller N2 but larger P3 effects than included participants, suggesting that excluded people invest less attention in unconscious conflict detection, but more in unconscious inhibition of impulsive responses. Together, these results suggest that exclusion causes people to rebalance attention allocation priorities for cognitive control according to a more flexible and adaptive strategy.

As social creatures, people have a fundamental need to feel socially accepted and maintain strong, stable social bonds^1^. However, this need to belong is often challenged by social exclusion, which is an aversive but prevalent phenomenon in daily life. When experiencing social exclusion, people would exhibit different approaches to cope with this challenge. Specifically, excluded people would demonstrate stronger prevention motivation, manifesting as increased threat vigilance and a more conservative response bias; while their counterparts, included people, would demonstrate stronger promotion motivation, manifesting as greater willingness to explore the outside world and a more risky response bias^2^,^3^.

Cognitive control, the collection of cognitive functions that allows people to control their behaviors in accordance with social norms, is essential for effective social connections^4^. Many studies have investigated how exclusion influences cognitive control, but the conclusions of these studies are inconsistent. Accordingly, exclusion has been reported to impair^5^,^6^,^7^, improve^8^,^9^, or have no significant effect^10^ on cognitive control. For instance, Jamieson *et al*.^6^ used an antisaccade task to explore the impacts of exclusion on inhibitory control and reported that excluded participants made more errors than included participants. On the contrary, Bernstein *et al*.^8^ reported that excluded participants were better able to distinguish a genuinely happy facial expression from a deceptive or false happy expression.

A unifying factor amongst these previous studies was the definition of cognitive control as a unitary concept. Recent research has proposed that, however, cognitive control involves two consecutive sub-processes: conflict detection and response implementation^11^,^12^. Moreover, evidence suggests that these two sub-processes depend on common but limited attentional resources, such that the effortful conflict detection process may deplete limited resources and reduce subsequent response implementation performance^13^,^14^. Therefore, conflicting results from previous studies could represent the measurement of different sub-processes underlying cognitive control. In support of this hypothesis, studies observing exclusion-mediated impairment of cognitive control primarily focused on the response implementation process^15^ or the combined outcomes of conflict detection and response implementation^6^,^7^, whereas studies reporting exclusion-mediated improvement of cognitive control studied the conflict detection process^8^,^9^.

The idea that exclusion can exert divergent effects on different sub-processes of cognitive control has been recently evaluated: using a Go/No-Go task, Otten and Jonas (2013) demonstrated that excluded participants exhibit a larger N2 but smaller P3 amplitude than included participants^4^. As the N2 and P3 indices represent conflict detection and inhibitory control, respectively^16^,^17^, these results suggest that exclusion affects sub-processes of cognitive control differently. Moreover, these data are consistent with the hypothesis that exclusion affects sub-processes of cognitive control in terms of attentional resources, wherein increased attention for conflict detection depletes resources for inhibitory control. In other words, exclusion might cause participants to deploy their attention in a “bottom-up” fashion^12^,^18^.

It is also important to note that existing studies have focused on the effect of exclusion on conscious cognitive control. While recent research has suggested the existence of unconscious cognitive control, such as unconscious inhibitory control^19^,^20^ and unconscious conflict adaptation^21^. For example, van Gaal *et al*. developed a masked Go/No-Go task wherein weakly masked Go/No-Go trials and strongly masked Go/No-Go trials were used to differentiate conscious and unconscious inhibitory control. In the weakly masked condition, a go or no-go prime was presented for 233 ms and followed by a metacontrast masking annulus for 17 ms, whereas these durations were reversed in the strongly masked condition. Inclusion of a strongly masked condition facilitates the direct measurement of reaction times (RTs) for unconscious no-go trials and thus the measurement of unconscious inhibitory capacity, with longer no-go RTs representing a stronger ability to suppress prepotent responses^20^,^22^. Using this masked Go/No-Go task, van Gaal *et al*. showed that responses in strongly masked no-go trials were slower than those in strongly masked go trials, validating the existence of unconscious inhibitory control. Moreover, other studies have reported that while both conscious and unconscious cognitive control activate the same prefrontal control networks^19^,^20^, the nature of this activation differs in strength, duration, and scope of neural activity^23^.

To date, only one behavioral study has investigated how exclusion influences conscious versus unconscious cognitive control^24^. DeWall *et al*.^24^ examined conscious and unconscious affective responses to social exclusion and reported that excluded participants had stronger unconscious emotional regulation than included participants, while no differences were found in conscious responses. These results suggest that exclusion differentially influences conscious and unconscious cognitive control. Since unconscious cognitive control processes come in very handy in real life, for example, the unconscious inhibition of some action tendencies probably helps us to avoid many embarrassing or socially inappropriate situations and might be crucial for adaptive goal-directed behaviour^20^,^25^, additional studies are therefore required to elucidate how exclusion affects the sub-processes of unconscious cognitive control, and whether these effects are similar or different to effects on conscious cognitive control.

In the present study, we sought to evaluate whether and how exclusion affects the sub-processes of conscious and unconscious cognitive control. To this end, we asked excluded and included participants to perform a masked Go/No-Go task as this task could distinguish between conscious and unconscious cognitive control^20^,^22^. We evaluated event-related potentials (ERPs) because they are particularly well-suited to the study of cognitive control and provide excellent temporal resolution. Previous studies have confirmed that in Go/No-Go paradigms (both traditional and masked Go/No-Go tasks), high conflict no-go trials typically elicit a larger negative potential with a maximum amplitude around 200 ms (N2) followed by a larger positive potential with a maximum around 300 ms (P3) than go trials^26^,^27^. Moreover, relative enhancements of N2 and P3 in no-go versus go trials (henceforth called the N2 effect and the P3 effect) are also useful indices for conflict detection and inhibitory control, respectively^4^,^27^. We therefore used the N2 and P3 components and enhancement effects to directly explore how social exclusion influences the sub-processes of cognitive control.

Building on previous studies^3^,^4^,^24^, we hypothesized that exclusion differently affects conscious and unconscious cognitive control. For conscious cognitive control, because excluded individuals are more vigilant to potential threats, we hypothesized that excluded individuals would deploy their attention in a “bottom-up” fashion^12^,^18^, utilizing more attentional resources for conscious conflict detection and fewer resources for the subsequent inhibitory process. Consequently, excluded participants were expected to demonstrate enhanced conflict detection (i.e., a larger N2 effect) and impaired inhibitory control (i.e., a smaller P3 effect) relative to included participants. For unconscious cognitive control, however, we speculated that this trend might differ: excluded individuals would show a conservative response bias and deploy their attention in a “top-down” fashion^12^,^18^,^24^, thus might prefer to treat vague or unconscious response conflicts as nonexistent; while included participants are more willing to explore the outside world and are likely to allocate resources for the detection of unconscious response conflicts. Therefore, we hypothesized that excluded participants would show diminished unconscious conflict detection (i.e., a smaller N2 effect) and increased unconscious inhibitory control (i.e., a larger P3 effect) relative to included participants.

## Results

### Behavioral data

#### Manipulation checks

Behavioral results are presented in Table 1 (all the data are freely available for download upon email request to the corresponding author). For the Need Threat scores, the results reveal lower scores for the exclusion (*M* = 2.99, *SD* = 1.08) than for the inclusion group (*M* = 5.24, *SD* = 0.75), *t* (34) = −7.28, *p* < 0.001, *d* = 2.42. These results suggest that the needs of excluded participants were threatened compared to those of the included participants, thereby confirming the effectiveness of the social exclusion manipulation.

For the PANAS scores, the results demonstrate that neither positive nor negative emotion scores differed significantly between the exclusion and inclusion groups (positive: *M* = 25.89, *SD* = 6.33 vs. *M* = 29.33, *SD* = 5.43, *t* (34) = −1.75, *p* = 0.089, *d* = 0.58; negative: *M* = 18.33, *SD* = 4.78 vs. *M* = 17.78, *SD* = 4.61, *t* (34) = 0.36, *p* = 0.725, *d* = 0.12). Consistent with previous studies, these results suggest that social exclusion did not cause explicit emotional responses^10^.

#### Awareness test

For the awareness test, the accuracies for both groups were at chance level (exclusion: *M* = 50.94%, *SD* = 0.08; inclusion: *M* = 47.94%, *SD* = 0.07), and *d*’s were not significantly different from zero (exclusion: *M* = −0.07, *SD* = 0.39, *t* (17) = −0.77, *p* = 0.450, *d* = 0.18; inclusion: *M* = 0.03, *SD* = 0.43, *t* (17) = 0.38, *p* = 0.740, *d* = 0.07). Therefore, the prime could not be perceived in the strongly masked condition, confirming the effectiveness of the masking procedure.

#### Masked Go/No-Go task

For the weakly masked condition, the ANOVA results on response accuracy only revealed a main effect of prime type, *F* (1, 34) = 80.17, *p* < 0.001, *η*^*2*^_*p*_ = 0.70, with lower accuracy for weakly masked no-go (*M* = 84.30%, *SD* = 0.01) than go trials (*M* = 99.20%, *SD* = 0.01). No other significant difference was observed. Moreover, no significant differences in the RTs for weakly masked go trials were found between exclusion (*M* = 456.95 ms, *SD* = 53.75) and inclusion (*M* = 462.62 ms, *SD* = 55.24) groups, *t* (34) = −0.31, *p* = 0.757, *d* = 0.10.

For the strongly masked condition, the mean response accuracy was above 99.00%; the main and interaction effects of group and prime type were not significant, *p’*s > 0.050. For the RTs, the results only revealed a main effect of prime type, *F* (1, 34) = 4.75, *p* = 0.036, *η*^*2*^_*p*_ = 0.12, with longer RTs for the strongly masked no-go (*M* = 391.28 ms, *SD* = 58.08) than go trials (*M* = 386.58 ms, *SD* = 60.36); therefore, it appears that unconscious inhibitory control existed in this study. No other significant difference was observed.

### ERP data

#### The effect of social exclusion on conscious cognitive control

The ANOVA results on N2 revealed a significant main effect of prime type, *F* (1, 34) = 25.44, *p* < 0.001, *η*^*2*^_*p*_ = 0.43, with more negative N2 for the weakly masked no-go (*M* = −1.71 μV, *SD* = 2.21) than go trials (*M* = −0.82 μV, *SD* = 2.02). We also found a significant interaction between group and prime type, *F* (1, 34) = 4.09, *p* = 0.051, *η*^*2*^_*p*_ = 0.11. Further analyses showed that, for the exclusion group, weakly masked no-go trials (*M* = −1.60 μV, *SD* = 3.12) evoked more negative N2 than go trials (*M* = 0.36 μV, *SD* = 2.85), *F* (1, 34) = 29.96, *p* < 0.001, *η*^*2*^_*p*_ = 0.42. For the inclusion group, weakly masked no-go trials (*M* = −1.81 μV, *SD* = 3.12) also evoked more negative N2 than go trials (*M* = −1.28 μV, *SD* = 2.85), *F* (1, 34) = 4.57, *p* = 0.040, *η*^*2*^_*p*_ = 0.12. No other significant difference was observed. More importantly, the *t*-test on the N2 effect showed that the N2 effect was larger for the exclusion (*M* = −1.24 μV, *SD* = 1.12) than the inclusion group (*M* = −0.53 μV, *SD* = 0.98), *t* (34) = −2.02, *p* = 0.051, *d* = 0.67, power (1–*β*) = 0.50.

The ANOVA on P3 revealed only a significant interaction between group and prime type, *F* (1, 34) = 4.37, *p* = 0.044, *η*^*2*^_*p*_ = 0.11. Further analyses showed that, for the inclusion group, weakly masked no-go trials (*M* = 9.00 μV, *SD* = 3.87) evoked a larger P3 than go trials (*M* = 7.21 μV, *SD* = 2.82), *F* (1, 34) = 5.68, *p* = 0.023, *η*^*2*^_*p*_ = 0.14. However, for the exclusion group, no significant differences were found between weakly masked no-go (*M* = 6.52 μV, *SD* = 6.02) and go trials (*M* = 6.95 μV, *SD* = 4.49), *F* (1, 34) = 0.33, *p* = 0.570, *η*^*2*^_*p*_ = 0.01. No other significant difference was observed. Importantly, the *t*-test on the P3 effect showed that the P3 effect was smaller for the exclusion (*M* = −0.43 μV, *SD* = 3.49) than the inclusion group (*M* = 1.79 μV, *SD* = 2.84), *t* (34) = −2.09, *p* = 0.044, *d* = 0.67, power (1–*β*) = 0.63.

#### The effect of social exclusion on unconscious cognitive control

The ANOVA results on N2 revealed a significant interaction between group and prime type, *F* (1, 34) = 6.69, *p* = 0.014, *η*^*2*^_*p*_ = 0.16. Further analyses showed that, for the inclusion group, strongly masked no-go trials (*M* = 0.16 μV, *SD* = 3.29) evoked more negative N2 than go trials (*M* = 1.11 μV, *SD* = 3.07), *F* (1, 34) = 9.69, *p* = 0.004, *η*^*2*^_*p*_ = 0.22. However, for the exclusion group, no significant differences were found between strongly masked no-go (*M* = 1.61 μV, *SD* = 2.22) and go trials (*M* = 1.44 μV, *SD* = 2.41), *F* (1, 34) = 0.30, *p* = 0.589, *η*^*2*^_*p*_ = 0.01. No other significant difference was observed. More importantly, the *t*-test on the N2 effect showed that the N2 effect was smaller for the exclusion (*M* = 0.17 μV, *SD* = 1.29) than the inclusion group (*M* = −0.95 μV, *SD* = 1.30), *t* (34) = 2.59, *p* = 0.014, *d* = 0.86, power (1 – *β*) = 0.71.

The ANOVA on P3 also only revealed a significant interaction between group and prime type, *F* (1, 34) = 4.89, *p* = 0.034, *η*^*2*^_*p*_ = 0.13. Further analyses showed that, for the exclusion group, strongly masked no-go trials (*M* = 4.81 μV, *SD* = 4.49) evoked a larger P3 than go trials (*M* = 4.25 μV, *SD* = 4.29), *F* (1, 34) = 4.15, *p* = 0.049, *η*^*2*^_*p*_ = 0.11. However, for the inclusion group, no significant differences were found between strongly masked no-go (*M* = 4.54 μV, *SD* = 3.36) and go trials (*M* = 4.83 μV, *SD* = 2.99), *F* (1, 34) = 1.19, *p* = 0.284, *η*^*2*^_*p*_ = 0.03. No other significant difference was observed. Importantly, the *t*-test on the P3 effect showed that the P3 effect was larger for the exclusion (*M* = 0.55 μV, *SD* = 1.15) than the inclusion group (*M* = −0.30 μV, *SD* = 1.15), *t* (34) = 2.21, *p* = 0.034, *d* = 0.74, power (1 – *β*) = 0.58.

## Discussion

The present study examined the effects of social exclusion on the sub-processes that underlie conscious and unconscious cognitive control. Consistent with our hypotheses, we found that exclusion differently affects the sub-processes underlying conscious and unconscious cognitive control. Specifically, for conscious cognitive control, excluded participants showed a larger N2 effect but smaller P3 effect than included participants. These results suggest that exclusion caused people to invest more attention on conscious conflict detection, but less to conscious inhibitory control. However, for unconscious cognitive control, excluded participants showed a smaller N2 effect but larger P3 effect than included participants. These results suggest that exclusion caused people to invest less attention in unconscious conflict detection, but more in unconscious inhibitory control.

We first found that exclusion exerted divergent effects on sub-processes of cognitive control, as evidenced by opposite N2 and P3 trends under both conscious and unconscious conditions. These results replicated the findings of Otten & Jonas (2013), who observed a larger conscious N2 effect and a smaller conscious P3 effect for excluded versus for included participants; however, our results also extended these findings to unconscious cognitive control. These findings can be explained by the hypothesis of limited attention capacity^14^, which proposes that all processes underlying cognitive control depend on common and limited attentional resources. In our current study, the N2 effect indexed conflict detection and the P3 effect indexed inhibitory control. Therefore, if conflict detection consumed many resources (larger N2 effect), few resources would remain for the following inhibitory control, causing it to be impaired (smaller P3 effect). Similarly, if conflict detection consumed few resources (smaller N2 effect), ample resources remained, leaving the following inhibitory control intact (larger P3 effect). These results also perfectly explain the observation of a null difference between behavioral levels in the current study. Specifically, behavioral results were the combined outcome of both conflict detection and inhibitory control processes: the ERPs effects counterbalanced one another and led to similar response patterns for excluded and included participants, respectively^4^.

A novel finding of our study was that the consciousness of cognitive control influences how exclusion affects the sub-processes of cognitive control. These results were partly consistent with previous studies^6^,^7^,^8^,^9^ and could be explained by the regulatory focus theory^2^,^3^,^28^. According to this theory, there are two broad motivational modes of goal pursuit: prevention and promotion. Prevention motivation represents the need for security (i.e., safety and protection), whereas promotion motivation represents the need for advancement (i.e., nourishment and growth). Prevention-focused individuals are vigilant for potential threat and show security-seeking responses. In contrast, promotion-focused individuals tend to make reward-seeking decisions and generally strive to achieve positive outcomes. Recent studies have demonstrated that excluded participants exhibit stronger prevention motivation while included participants exhibit stronger promotion motivation^3^. Accordingly in our study, for conscious cognitive control, excluded participants prioritized more attentional resources for conscious conflict detection (as these overt conflicts might represent potential threat) and consequently utilized fewer resources for inhibitory control, leading to a large N2 effect and a small P3 effect. However, for unconscious cognitive control, excluded participants might conservatively disregard unapparent conflicts and therefore invested few resources in conflict detection, leaving ample resources for subsequent inhibitory control, and this resulted in a small N2 effect and a large P3 effect. While included participants could be more risky and willing to explore the unconscious conflicts, thus invested more resources in conflict detection and left fewer resources for subsequent inhibitory control, leading to a large N2 effect and a small P3 effect. This allocation strategy thus contributed to smaller N2 and larger P3 effect for excluded participants than for included participants.

Taken together, these results suggest that exclusion caused participants to adopt different strategies of attention allocation for conscious and unconscious cognitive control. When task demands were explicit (weakly masked, conscious condition), excluded individuals would prefer to adopt a reactive control strategy and allocated their attention in a “bottom-up” fashion^12^,^18^, heightening conflict monitoring (N2) at the expense of open monitoring (P3). Nevertheless, if task demands were implicit (strongly masked, unconscious condition), they would adopt a proactive control strategy and allocated their attention in a “top-down” fashion^12^,^18^, heightening open monitoring. In a word, after exclusion, attention is flexibly applied across these two conditions.

Then the subsequent question was why excluded participants adopted these different strategies. To answer this question, it is helpful to reemphasize the fundamental role of the need to belong^1^. Given the importance of the need to belong, excluded people would like to make new social connections, but above all they want to make sure that they will not suffer through being rejected again^3^. Consequently, when potential threat (e.g. response conflict) is obvious, they would deploy many resources to detect it and make a quick response. However, when the threat is unapparent or trivial, they would disregard it and allocate more resources to ensure the performance on more important response implementation. Since the successful inhibition of inappropriate behavior is essential for effective social connection and interaction^4^, the latter attention allocation strategy for unconscious cognitive control might be helpful for excluded participants to reconnect with others. Taken together, we conclude that following social exclusion, individuals adopt a more flexible and adaptive strategy for attention allocation in cognitive control: individuals prioritize the detection of obvious threats so as to make a quick response, whereas less obvious threats are disregarded and priority is given to inhibitory control. As the attentional resources are limited, this strategy might be the most adaptive one for excluded people because they could centralize resources to reduce the possibility of future exclusion while increase the likelihood of future inclusion.

Previously, many studies have explored the influences of exclusion on cognitive control but reported conflicting results^5^,^6^,^7^,^8^,^9^. By dividing cognitive control into multiple sub-processes, our results as well as those of Otten and Jonas (2013) have been able to reconcile the controversy over the impact of exclusion on cognitive control (impair, improve, or have no significant effect). More importantly, by adopting the masked Go/No-Go task, our study was able to distinguish between conscious and unconscious cognitive control and present an integrated picture of how exclusion influences cognitive control. After experiencing social exclusion, people appear to actively rebalance priorities of attention allocation in cognitive control with a more flexible and adaptive strategy, rather than passively bear adverse consequences. Apart from these theoretical contributions, the current results are also relevant from a practical perspective: while existing studies primarily focus on the how to relieve the negative impacts of social exclusion^29^,^30^, our results suggest that future studies should investigate the benefits of exclusion for cognitive control. Indeed, some studies have shown that exclusion promotes creative thought^31^ and the ability to manage others’ emotion^32^.

Notably, the present study was limited by several factors. First, we failed to measure the motivations for promotion and prevention after the exclusion and inclusion manipulations. Therefore, our explanations regarding motivation are speculative to some extent. Nevertheless, as a recent study has directly demonstrated that excluded participants exerted prevention motivation and included participants exerted promotion motivation^3^, we thought our speculation was reasonable. However, Future studies should directly measure the motivations for promotion and prevention after the exclusion manipulation and examine whether motivation mediates the relationship between exclusion and cognitive control. Second, we did not consider the potential influence of individual differences. Studies have shown that individual differences such as rejection sensitivity^33^ and anxiety^24^ can influence the relationship between exclusion and cognitive control. Future studies should take this issue into consideration. Third, as we reported in our results, statistical power (1 – *β*) ranged between 0.50 and 0.71 in the current study, which was smaller than the acceptable power (0.8). Moreover, in order to detect medium-size effects (size *d* = 0.5) at the desired significance levels of *α* = *β* = 0.5, data should have been collected from a sample of at least *N* = 210 participants^34^. Consequently, a small sample size was a major limitation that should be addressed in future studies. Fourth, as we adopted the Cyberball task to manipulate exclusion, a pure control condition was not included, and excluded participants were only compared to included participants. This made condition differences difficult to interpret because a difference could have been due to an effect among excluded participants, an effect among included participants, or both. Therefore, future studies should try to avoid this problem. Fifth, although our current results could show us some information about how exclusion influenced attention, this was insufficient as attention contained different aspects (alerting, orienting, and conflict resolution), thus more studies are needed to explore how exclusion affects these different attentional networks^35^,^36^.

## Conclusion

The present study demonstrated that exclusion differently affects the sub-processes underlying conscious and unconscious cognitive control. Specifically, for conscious cognitive control, exclusion caused individuals to invest more attention in the detection of conscious response conflict, but less in the actual conscious inhibition of impulsive responses. For unconscious cognitive control, exclusion caused individuals to invest less attention in the detection of unconscious response conflict, but more in the actual unconscious inhibition of impulsive responses. Together, these results suggest that exclusion causes people to rebalance the attention allocation priorities of cognitive control with a more flexible and adaptive strategy.

## Methods

### Participants

A total of 36 Chinese university students (30 females) aged 18–23 years (*M* = 20.69 years, *SD* = 1.24) participated in the present study. Each participant was compensated with 40 RMB (approximately $6.50 USD). Participants were randomly assigned to either the inclusion group or the exclusion group. After completing a masked Go/No-Go task, participants were debriefed and dismissed. We only recruited participants who had never taken part in similar experiments involving social interactions with other players. All participants gave informed consent prior to the initiation of the experiment, and all study procedures were conducted in accordance with the Declaration of Helsinki and approved by the Ethics Committee of the School of Psychology, Southwest University.

## Materials and Procedure

### Cyberball game

The current study utilized a virtual ball-tossing game called Cyberball in order to manipulate social exclusion. In the Cyberball paradigm, participants played a virtual toss game with two other players that they did not know and did not expect to meet^37^. We manipulated the degree of social exclusion and inclusion by varying the number of times participants received the ball from the other players. Participants in the inclusion group received the ball in approximately one-third of the total throws (40 total throws), while participants in the exclusion group only received the ball twice at the beginning of the game^38^.

### Need Threat Scale

After completion of the Cyberball game, participants completed the 20-item Need Threat Scale^39^. This scale asked participants to self-assess their level of satisfaction for feelings of belonging, self-esteem, meaningful existence, and control during the game on a seven-point scale (1 = “do not agree” to 7 = “agree”; *α* = 0.92). Lower scores represented an increased perceived threat to social needs and indicated the effectiveness of the social exclusion manipulation.

### Positive and Negative Affect Schedule (PANAS)

Participants also completed the 20-item PANAS^40^. The PANAS includes ten items assessing positive emotions (e.g., interested, excited) and ten items assessing negative emotions (e.g., irritable, ashamed). Participants were instructed to self-assess their current emotional state on a five-point scale (1 = “very slightly or not at all” to 5 = “extremely”; *α* = 0.97).

### Masked Go/No-Go task

**Figure 1:**
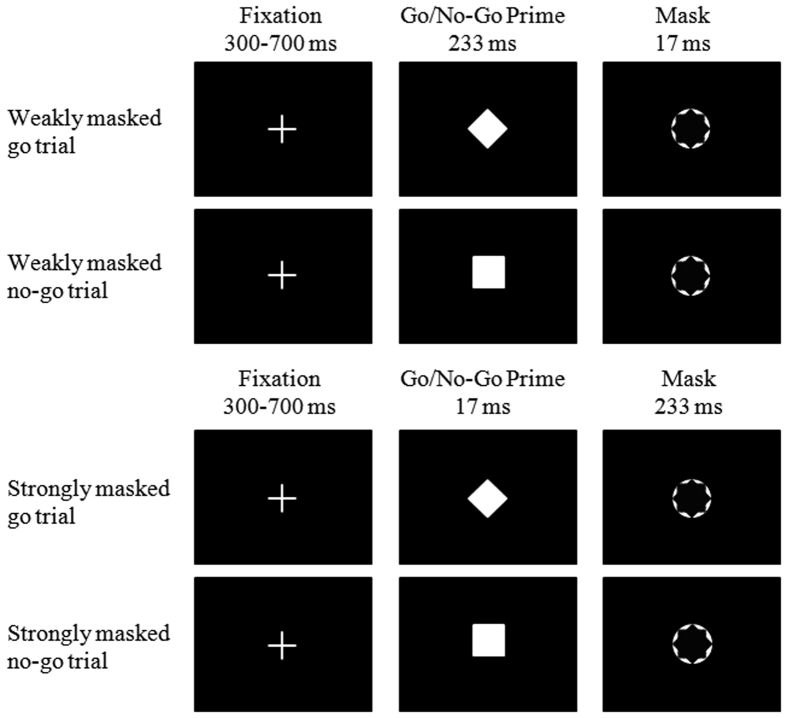
The masked Go/No-Go task used in the present study.

The masked Go/No-Go task was adapted from van Gaal *et al*. and consisted of weakly and strongly masked go/no-go trials (Fig. 1). In the weakly masked condition, a fixation point (300–700 ms) appeared and was followed by a go or no-go prime (visual angle 0.47° × 0.47°) of relatively long duration (233 ms) and a briefly presented metacontrast masking annulus (visual angle of 0.8°) for a short duration (17 ms)^22^. In the strongly masked condition, the durations of the prime and annulus were 17 and 233 ms, respectively. We used the annulus as a metacontrast mask because it strongly reduced stimulus visibility^41^. The between-trial interval was 1500 ms.

Participants viewed the display from a fixed distance of 60 cm. They were instructed to respond to a white annulus (response signal under strongly masked condition) as quickly as possible by pressing the “M” key on a standard keyboard with their right index finger, while withhold their response when a white square (no-go signal) preceded the annulus. However, when a white diamond (go signal) preceded the annulus, they were instructed to respond as quickly as possible by pressing the “M” key.

There were three blocks containing 108 trials each for a total of 324 trials, with 81 trials for each condition (i.e., weakly masked go trial, weakly masked no-go trial, strongly masked go trial, and strongly masked no-go trial). The presentation of go/no-go prime was displayed in a random order. The stimulus used as the no-go signal (square or diamond) was counterbalanced across participants. Before the experiment began, participants completed a practice block of 40 trials to ensure that they understood the task instructions.

### The awareness test

The prerequisite of the masked Go/No-Go task was the effectiveness of its masking procedure. Therefore, to assess whether participants were truly unaware of the strongly masked prime, an alternative forced-choice discrimination task was added, which included 80 strongly masked trials after the formal task. Participants were asked to press “F” or “J” depending on the shape (square or diamond) that flashed before the annulus in the trial. They were informed that response time was not important and were asked to respond as accurately as possible.

### EEG recordings and data reduction

Brain electrical activity was recorded at 64 scalp sites using tin electrodes mounted in an elasticcap (Brain Product, Munich, Germany), with references on the left and right mastoids and a ground electrode on the medial frontal aspect. The vertical electrooculograms (EOGs) were recorded supra- and infra-orbitally at the right eye. The horizontal EOG was recorded from the left versus the right orbital rim. The EEG and EOGs were amplified using a 0.05–100 Hz bandpass and continuously digitized at 500 Hz/channel. Inter-electrode impedance was maintained below 5 kΩ. Offline, the data were referenced to the average of the left and right mastoids (average mastoid reference), and a bandpass filter of 0.1–40 Hz was applied. Eye movement artifacts (such as eye movements and blinking) were excluded offline. Trials contaminated with artifacts due to amplifier clipping and peak-to-peak deflection exceeding ± 80 μV were excluded from the average. Only trials with correct responses were analyzed. About 6.8% of total trials were excluded from following analyses. The continuous recording was divided into 700-ms epochs for each trial, beginning 200 ms before the go/no-go signal onset.

**Figure 2:**
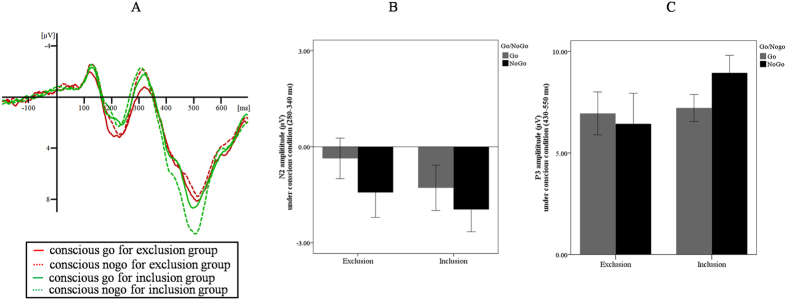
ERP results for the weakly masked condition: (**A**) The averaged ERP for the exclusion and inclusion groups; (**B**) N2 amplitudes under different contexts; and (**C**) P3 amplitudes under different contexts. Errors bars represent standard errors.

**Figure 3:**
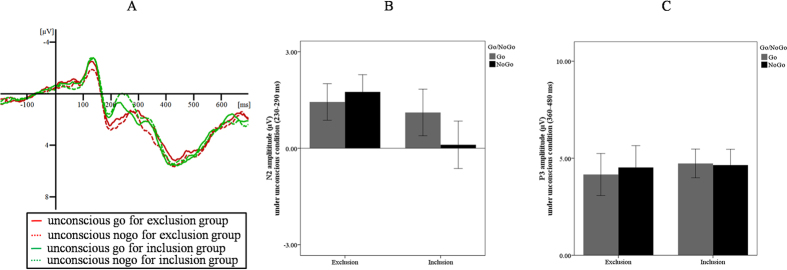
ERP results for the strongly masked condition: (**A**) The averaged ERP for the exclusion and inclusion groups; (**B**) N2 amplitudes under different contexts; and (**C**) P3 amplitudes under different contexts. Errors bars represent standard errors.

To increase the signal-to-noise ratio, we created a region of interest for the N2 and P3 components consisting of several fronto-central electrodes (Fz, C1, C2, Cz, FC1, FC2, and FCz). This region of interest was selected based on previous studies^19^,^23^,^27^, the waveforms and the voltage scalp maps (the spatio-temporal differences between the processing of go and no-go trials, see supplementary information) obtained in our current study. Consistent with previous research, mean amplitudes of specific ERP deflections were measured for different time intervals^19^. The time windows of the N2 and P3 components of obtained average waveforms were also based on previous studies^19^,^23^,^27^, the waveforms and the voltage scalp maps obtained in our current study. Consequently, for the weakly masked condition, the N2 component interval was 280–340 ms and the P3 component interval was 430–550 ms; for the strongly masked condition, the N2 component interval was 230–290 ms and the P3 component interval was 360–480 ms (Figs 2 and 3).

### Data analysis

#### Behavioral analysis

First, in order to the test whether the exclusion manipulation was effective, the Need Threat Scale and PANAS scores were separately analyzed with an independent sample *t*-test between exclusion and inclusion groups. Second, for the awareness test, a one-sample *t*-test was performed on the *d*’ scores (tested against 0) for the exclusion and inclusion groups, respectively. Third, for the masked Go/No-Go task, the key dependent variables were accuracy and reaction times. Only correct responses between 100 and 1200 ms were analyzed^22^. For the weakly masked condition, accuracy for go/no-go trials was first analyzed using a group (exclusion, inclusion) × prime type (weakly masked go trial vs. weakly masked no-go trial) ANOVA; RTs for go trials in exclusion and inclusion groups were analyzed using an independent sample *t*-test. For the strongly masked condition, accuracy (i.e., the response rate, as participants were asked to respond to annulus when they could not perceive the unconscious go/no-go signal) and RTs were separately analyzed using a group (exclusion, inclusion) × prime type (strongly masked go trial vs. strongly masked no-go trial) ANOVA.

#### ERP analysis

For both the weakly masked and strongly masked conditions, N2 and P3 amplitudes were first analyzed using a group (exclusion, inclusion) × prime type (weakly masked go trial vs. weakly masked no-go trial) ANOVA. Then, the N2 and P3 effect (difference waves of no-go minus go conditions) for exclusion and inclusion groups were separately analyzed using an independent sample *t*-test.

## Additional Information

**How to cite this article**: Xu, M. *et al*. Social exclusion modulates priorities of attention allocation in cognitive control. *Sci. Rep*. **6**, 31282; doi: 10.1038/srep31282 (2016).

## Supplementary Material

Supplementary Information

## Figures and Tables

**Table 1:** Means and standard deviations of Need Threat Scale and PANAS scores and behavioral results (reaction times [RTs] and accuracy) for masked Go/No-Go task.

	Exclusion	Inclusion
	*M* (*SD*)	*M* (*SD*)
Need threats	2.99 (1.08)	5.24 (0.76)
Positive	25.89 (6.33)	29.33 (5.43)
Negative	18.33 (4.78)	17.78 (4.61)
Behavior results		
Weakly masked trials		
Go RT	456.95 (53.75)	462.62 (55.24)
Go accuracy	0.99 (0.02)	0.99 (0.01)
No-go accuracy	0.85 (0.06)	0.84 (0.13)
Strongly masked trials		
Go RT	382.84 (63.11)	390.32 (57.60)
No-go RT	389.05 (60.44)	393.51 (55.60)
Go accuracy	0.99 (0.01)	0.99 (0.02)
No-go accuracy	0.99 (0.01)	0.99 (0.01)
