# Supplementary material for: Social exclusion modulates priorities of attention allocation in cognitive control
Source: Sci Rep. 2016 Aug 11;6:31282. doi: 10.1038/srep31282 (PMC4980633; doi:10.1038/srep31282)
Supplement: Supplementary Information [file srep31282-s1.doc]

**Social exclusion** **modulates** **priorities of attention allocation in cognitive control**

Mengsi Xu^1,2^, Zhiai Li^3^, Liuting Diao^1,2^, Lijie Zhang^1,2^, Jiajin Yuan^1,2^, Cody Ding^1,2,4^, Dong Yang^1,2*^

^1^School of Psychology, Southwest University, Chongqing, China

^2^Key Laboratory of Cognition and Personality (Southwest University), Ministry of Education, Chongqing, China

^3^The School of Psychology and Cognitive Science, East China Normal University, Shanghai, China

^4^ University of Missouri-St. Louis, St. Louis, USA

* **Corresponding author**

E-mail: [yangd@swu.edu.cn](mailto:yangd@swu.edu.cn)


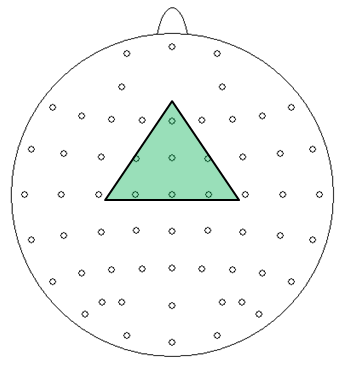


**Supplementary Figure S1.** Channel layout used for statistical analysis. Clusters of channels used for statistical analysis (Fz, FCz, FC1, FC2, Cz, C1, C2) are highlighted in triangle. In the statistical analyses here, we used a grand average and selection of a time window, however, this window might do not capture the peaks, when present, for each individual subject.

A


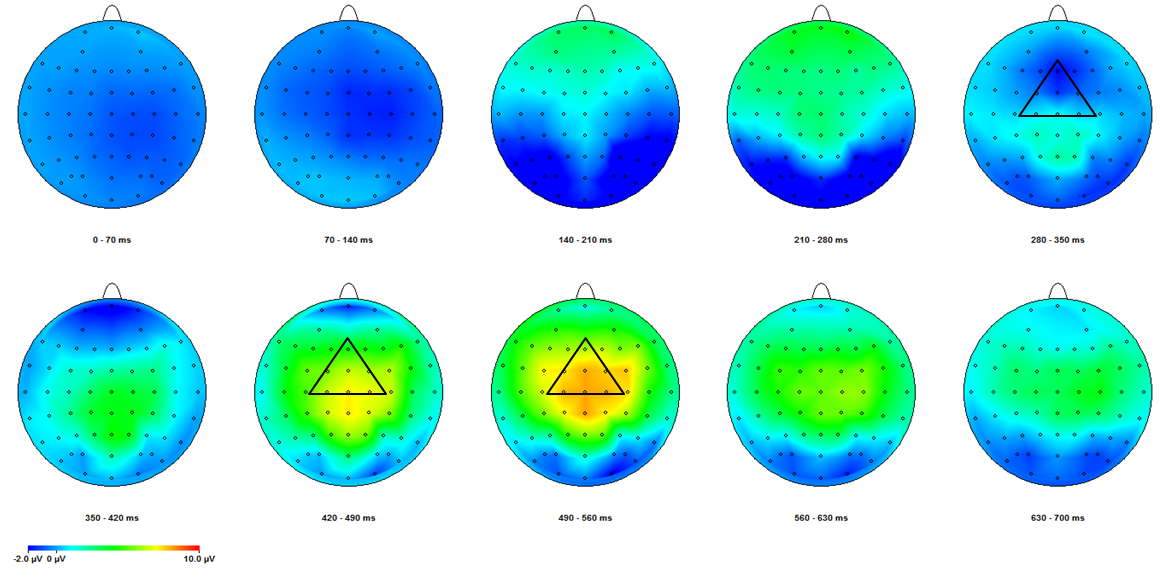


B


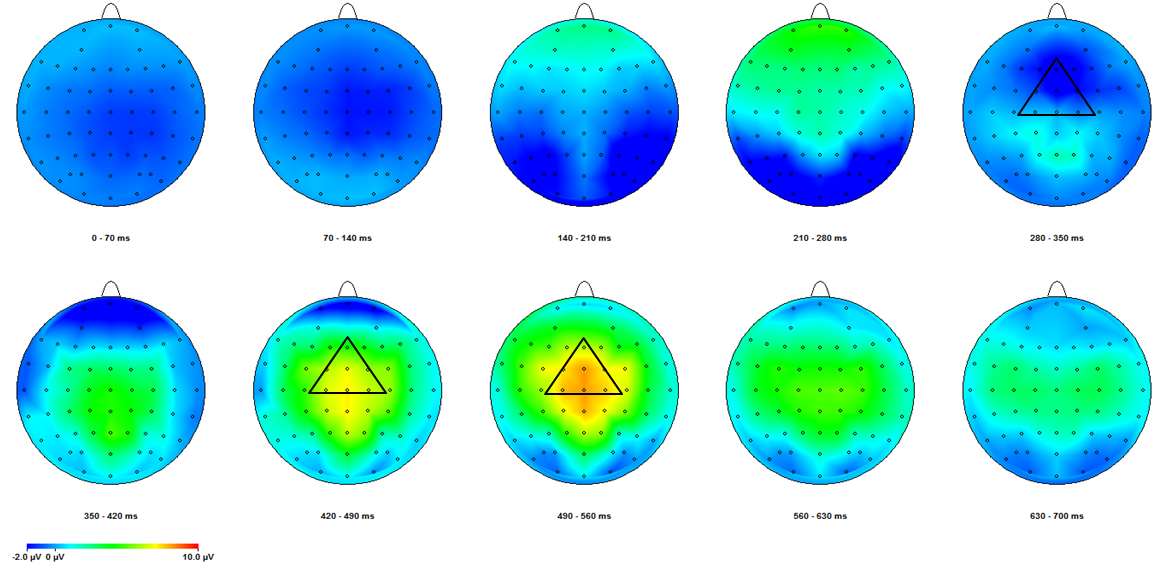


C


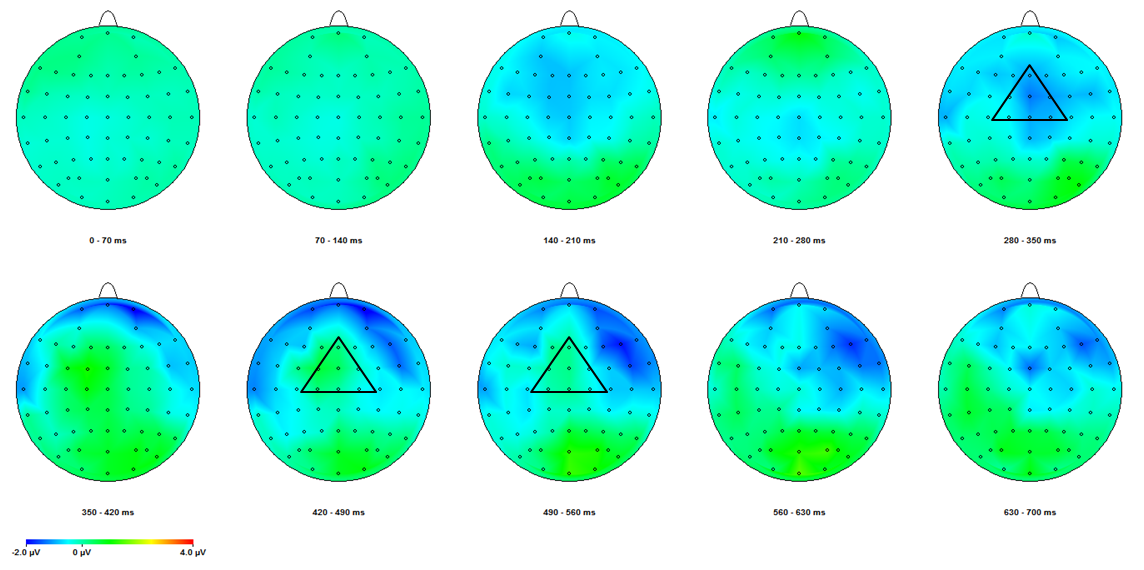


**Supplementary Figure S2.** A: Voltage scalp maps showing the processing of conscious go trials for exclusion group; B: Voltage scalp maps showing the processing of conscious no-go trials for exclusion group; and C: Voltage scalp maps showing the spatio-temporal differences between the processing of conscious no-go trials and conscious go trials for exclusion group. The electrodes used for statistical analyses are highlighted in triangle.

A


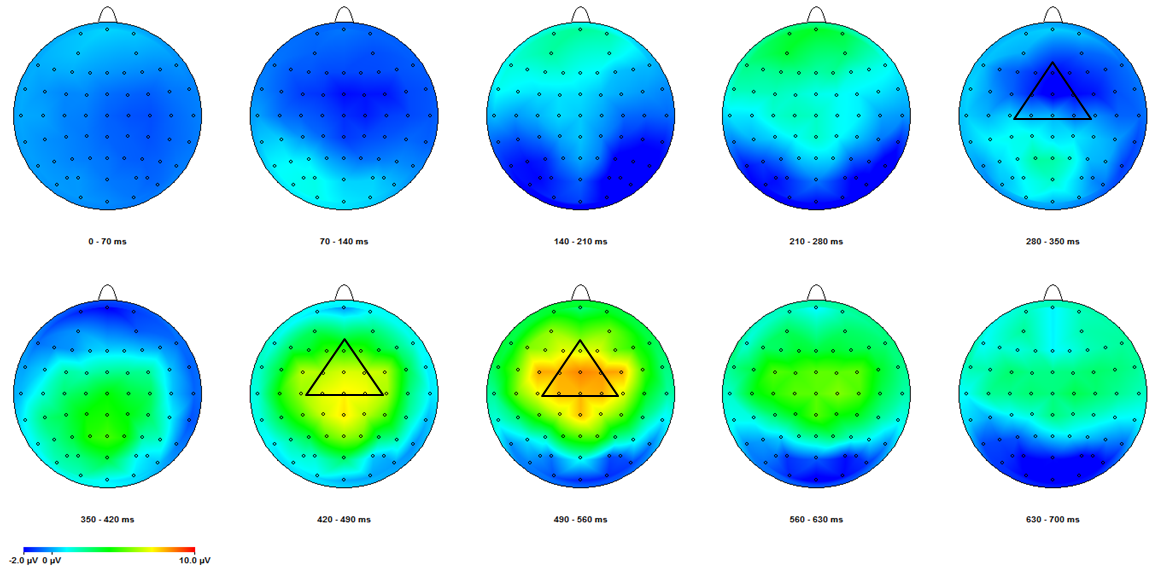


B


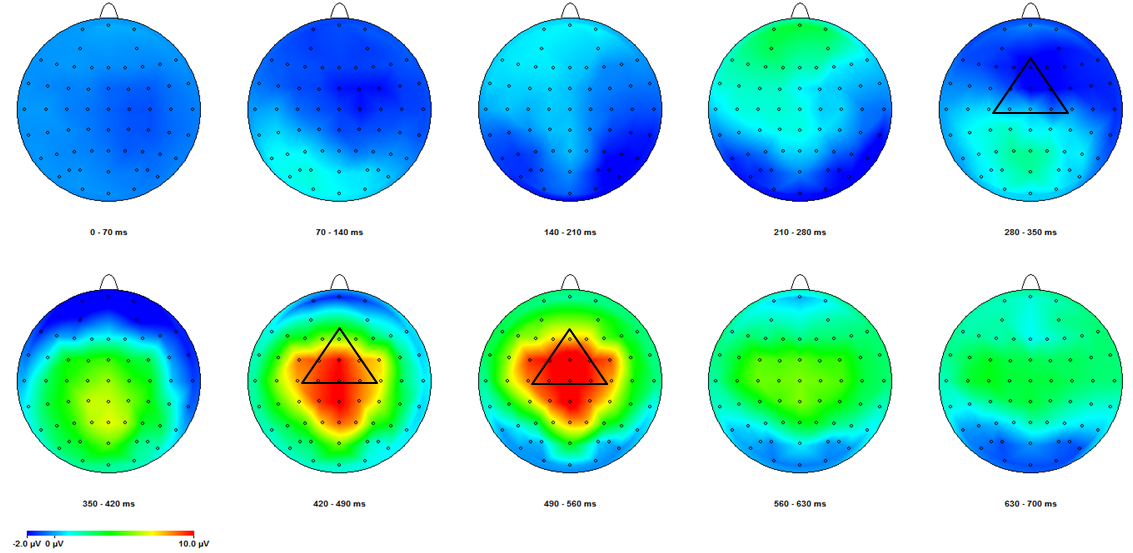


C


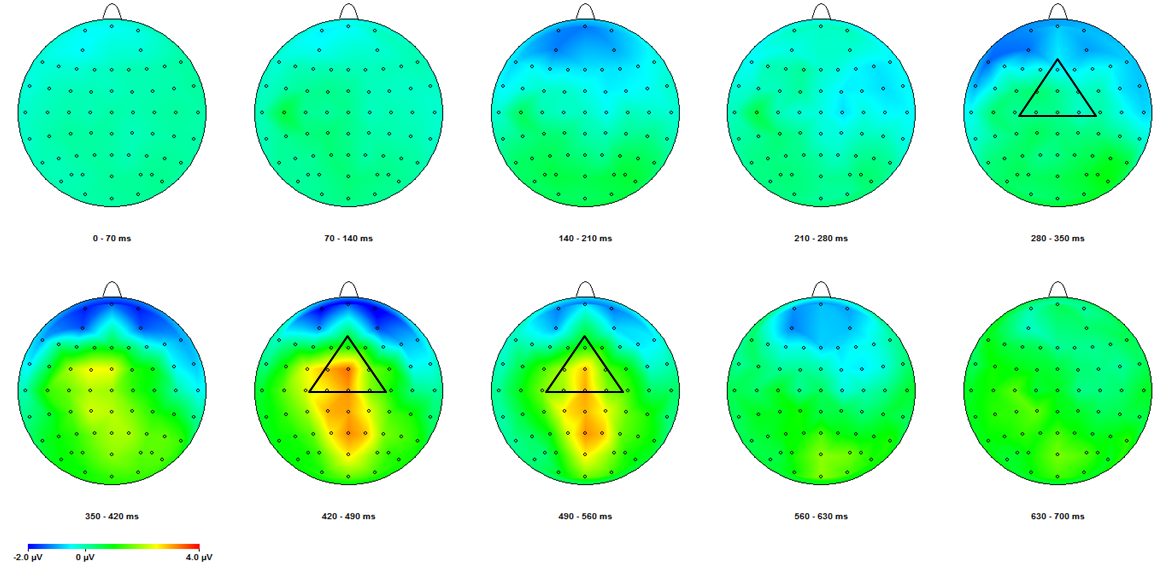


**Supplementary Figure S3.** A: Voltage scalp maps showing the processing of conscious go trials for inclusion group; B: Voltage scalp maps showing the processing of conscious no-go trials for inclusion group; and C: Voltage scalp maps showing the spatio-temporal differences between the processing of conscious no-go trials and conscious go trials for inclusion group. The electrodes used for statistical analyses are highlighted in triangle.

A


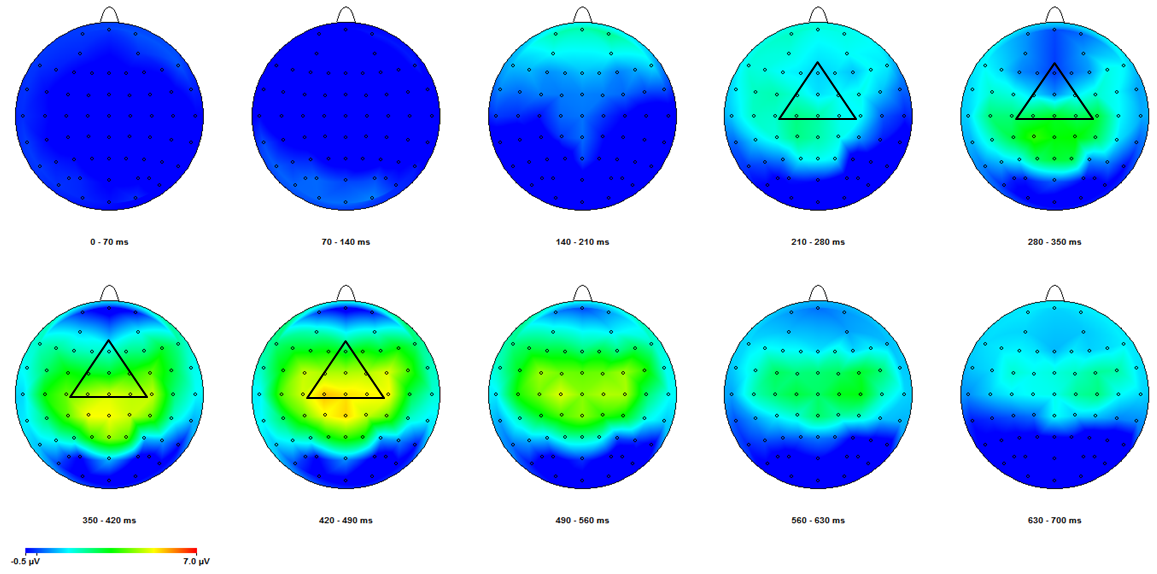


B


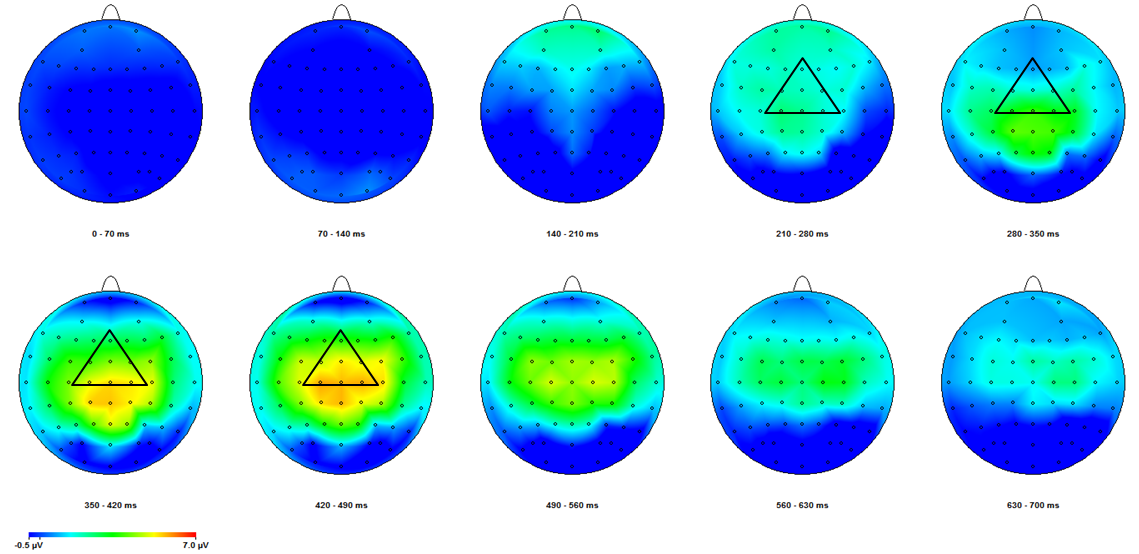


C


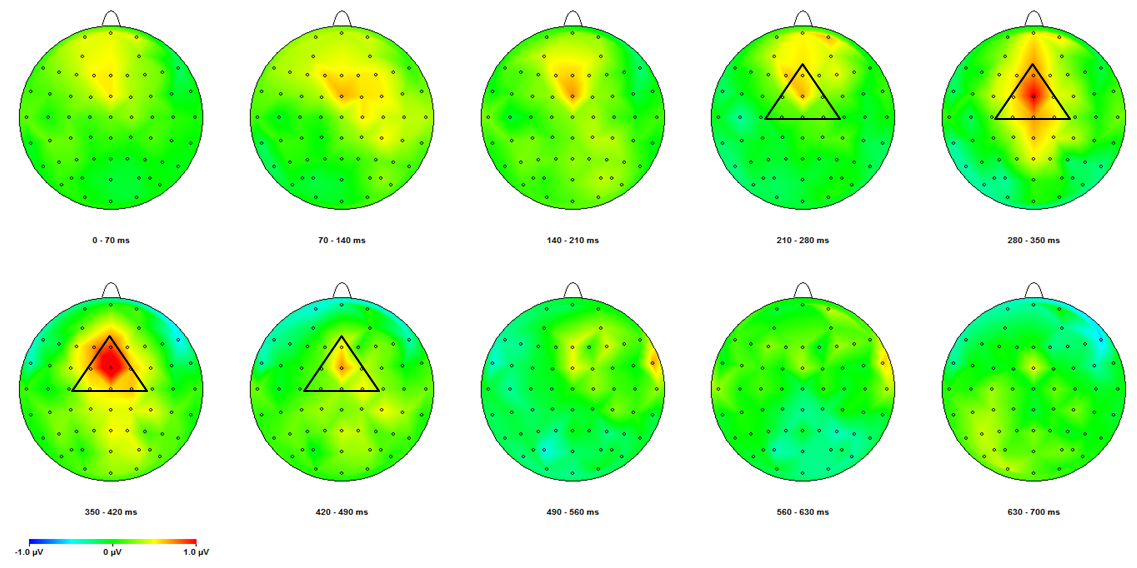


**Supplementary Figure S4.** A: Voltage scalp maps showing the processing of unconscious go trials for exclusion group; B: Voltage scalp maps showing the processing of unconscious no-go trials for exclusion group; and C: Voltage scalp maps showing the spatio-temporal differences between the processing of unconscious no-go trials and unconscious go trials for exclusion group. The electrodes used for statistical analyses are highlighted in triangle.

A


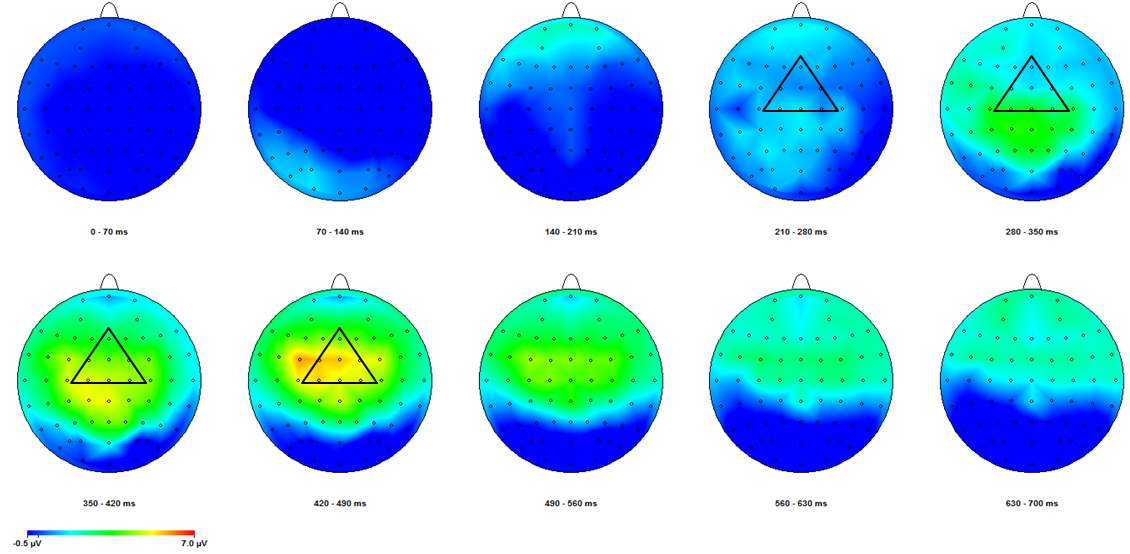


B


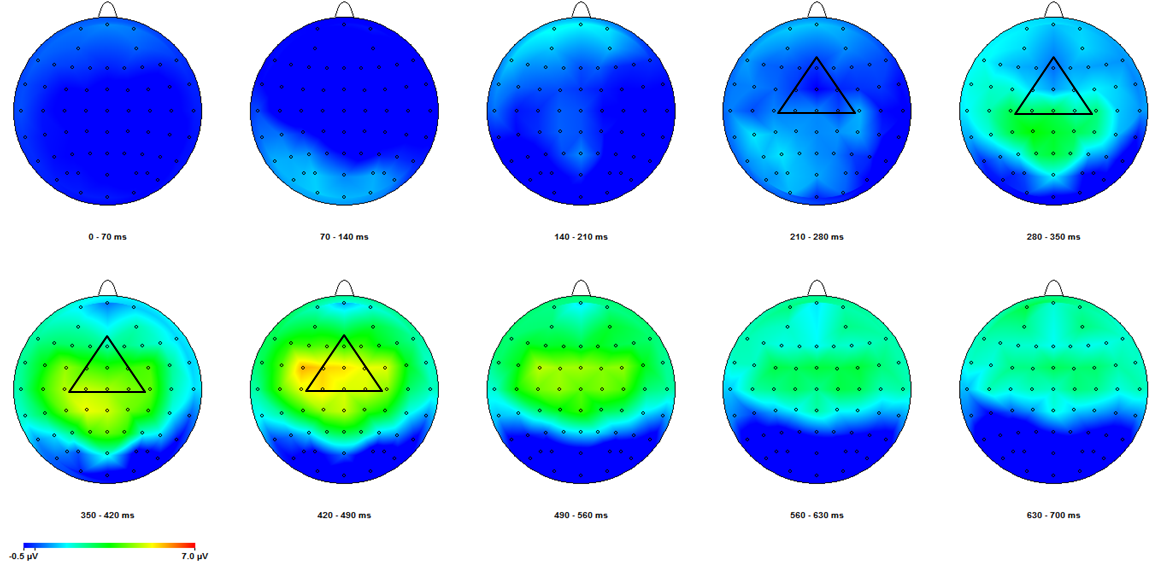


C


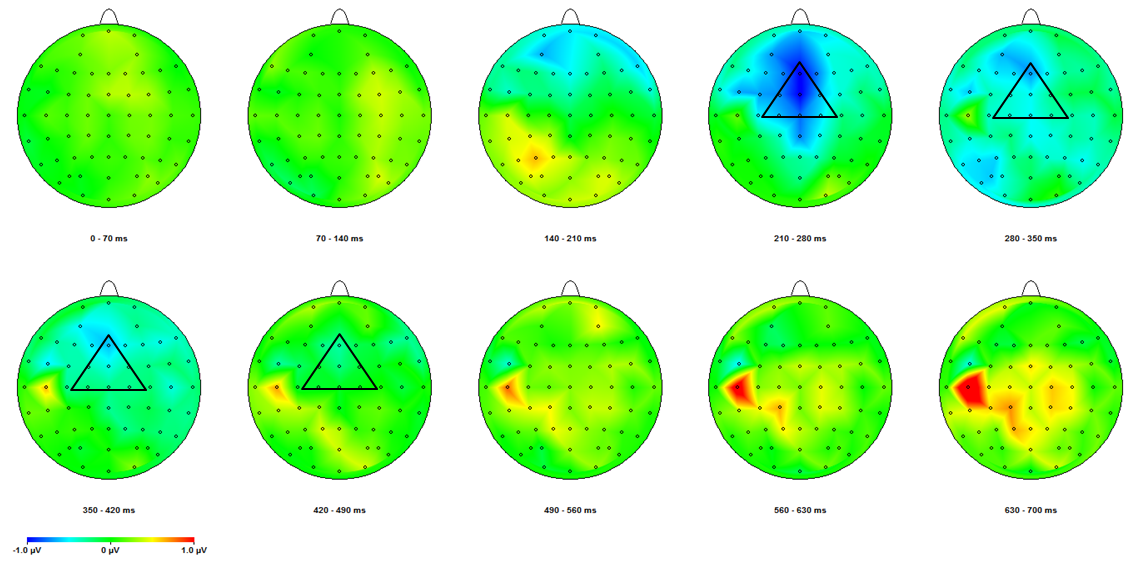


**Supplementary Figure S5.** A: Voltage scalp maps showing the processing of unconscious go trials for inclusion group; B: Voltage scalp maps showing the processing of unconscious no-go trials for inclusion group; and C: Voltage scalp maps showing the spatio-temporal differences between the processing of unconscious no-go trials and unconscious go trials for inclusion group. The electrodes used for statistical analyses are highlighted in triangle.

**Table S1**. Average numbers of good trials (means and standard deviations) for each condition across different groups.

|  | UG | UNG | CG | CNG |
| --- | --- | --- | --- | --- |
| Exclusion | 78.89 (3.05) | 78.17 (3.87) | 77.83 (3.76) | 66.39 (10.56) |
| Inclusion | 77.89 (2.95) | 77.67 (3.05) | 78.56 (1.95) | 68.50 (4.97) |

Note: UG: unconscious-go; UNG: unconscious-nogo; CG: conscious-go; CNG: conscious-nogo.

**Supplementary Analyses T1.** Analyses using the Need Threat Scale scores as a covariate in association with the effects of conscious N2 (N2 differences between conscious no-go and go conditions), conscious P3 (P3 differences between conscious no-go and go conditions), unconscious N2 (N2 differences between unconscious no-go and go conditions), and unconscious P3 (P3 differences between unconscious no-go and go conditions), respectively.

1. Conscious N2 effect

| Predictors | B | Std. Error | Beta (*β*) | t | Sig. |
| --- | --- | --- | --- | --- | --- |
| Constant | -.223 | .774 |  | -.288 | .777 |
| Need Threat Scale scores | -.340 | .244 | -.329 | -1.392 | .183 |
| R = .329; R^2^ = .108; Ajusted R^2^ = .052; *F* = 1.938; *p* = .183 | | | | | |

2. Conscious P3 effect

| Predictors | B | Std. Error | Beta (*β*) | t | Sig. |
| --- | --- | --- | --- | --- | --- |
| Constant | -.1.184 | 2.771 |  | -.427 | .675 |
| Need Threat Scale scores | .221 | .876 | .063 | .253 | .804 |
| R = .063; R^2^ = .004; Ajusted R^2^ = -.058; *F* = .064; *p* = .804 | | | | | |

3. Unconscious N2 effect

| Predictors | B | Std. Error | Beta (*β*) | t | Sig. |
| --- | --- | --- | --- | --- | --- |
| Constant | -.102 | .946 |  | -.108 | .916 |
| Need Threat Scale scores | -.090 | .299 | .075 | .301 | .768 |
| R = .075; R^2^ = .006; Ajusted R^2^ = -.057; *F* = .090; *p* = .768 | | | | | |

4. Unconscious P3 effect

| Predictors | B | Std. Error | Beta (*β*) | t | Sig. |
| --- | --- | --- | --- | --- | --- |
| Constant | .356 | .843 |  | .423 | .678 |
| Need Threat Scale scores | .066 | .266 | .062 | .248 | .807 |
| R = .062; R^2^ = .004; Ajusted R^2^ = -.058; *F* = .062; *p* = .807 | | | | | |
